# Environmental control of asexual reproduction and somatic growth of *Aurelia* spp. (Cnidaria, Scyphozoa) polyps from the Adriatic Sea

**DOI:** 10.1371/journal.pone.0178482

**Published:** 2017-06-14

**Authors:** Nathan Hubot, Cathy H. Lucas, Stefano Piraino

**Affiliations:** 1 Brussels Free University, Brussels, Belgium; 2 National Oceanography Centre Southampton, University of Southampton, Southampton, United Kingdom; 3 Dipartimento di Scienze e Tecnologie Biologiche ed Ambientali (DiSTeBA), University of Salento, Lecce, Italy; 4 Consorzio Nazionale Interuniversitario per le Scienze del Mare, Roma, Italy; University of California Irvine, UNITED STATES

## Abstract

Polyps of two moon jellyfish species, *Aurelia coerulea* and *A*. *relicta*, from two Adriatic Sea coastal habitats were incubated under multiple combinations of temperature (14, 21°C), salinity (24, 37 ppt) and food regime (9.3, 18.6, 27.9 μg C ind^−1^ week^−1^) to comparatively assess how these factors may influence major asexual reproduction processes in the two species. Both species exhibited a shared pattern of budding mode (Directly Budded Polyps: DBP; Stolonal Budded Polyps: SBP), with DBP favoured under low food supply (9.3 μg C ind ^−1^ week^−1^) and low temperature (14°C), and SBP dominant under high temperature (21°C). However, *A*. *coerulea* showed an overall higher productivity than *A*. *relicta*, in terms of budding and podocyst production rates. Further, *A*. *coerule*a exhibited a wide physiological plasticity across different temperatures and salinities as typical adaptation to ecological features of transitional coastal habitats. This may support the hypothesis that the invasion of *A*. *coerulea* across coastal habitats worldwide has been driven by shellfish aquaculture, with scyphistoma polyps and resting stages commonly found on bivalve shells. On the contrary, *A*. *relicta* appears to be strongly stenovalent, with cold, marine environmental optimal preferences (salinity 37 ppt, T ranging 14–19°C), corroborating the hypothesis of endemicity within the highly peculiar habitat of the Mljet lake. By exposing *A*. *relicta* polyps to slightly higher temperature (21°C), a previously unknown developmental mode was observed, by the sessile polyp regressing into a dispersive, temporarily unattached and tentacle-less, non-feeding stage. This may allow *A*. *relicta* polyps to escape climatic anomalies associated to warming of surface layers and deepening of isotherms, by moving into deeper, colder layers. Overall, investigations on species-specific eco-physiological and ontogenetic potentials of polyp stages may contribute to clarify the biogeographic distribution of jellyfish and the phylogenetic relationships among evolutionary related sister clades.

## Introduction

Jellyfish outbreaks have been attributed to natural and/or anthropogenic causes, including climate change [1–4]. In several coastal ecosystems jellyfish may produce major impacts to human activities and ecosystem services, including significant losses in different economic sectors [2,5–7] and ecological and societal benefits [8,9]. In this context, understanding the biological mechanisms and related environmental envelopes underlying jellyfish outbreaks is crucial to predict and mitigate impacts of recurrent bloom events. Most species of Scyphozoa have a polymorphic life cycle involving a short-living larval stage (planula), a benthic asexual post-larval stage (polyp), and a pelagic sexual stage (medusa). For these species, the occurrence of jellyfish outbreaks is thought to be directly linked to the ecological success of the benthic stage [10,11].

The moon jellyfish *Aurelia aurita* (Linnaeus 1758) had been previously described as a nearly cosmopolitan ecological generalist [12]. Phylogenetic studies later identified at least 3 valid morphospecies (*A*. *aurita*, *A*. *limbata*, *A*. *labiata*) plus 13 additional molecular species [13–15]. Overall, the *Aurelia* spp. taxon can be regarded as the most widely distributed scyphozoan group, a species-complex composed of numerous locally adapted species mainly found in coastal waters [15–19].

*Aurelia* spp. polyps can asexually multiply (mainly by polyp budding), produce resting stages (podocyst formation), or advance the life cycle by the production of juvenile medusae (ephyrae) via strobilation [20]. Previous studies showed these ontogenetic processes are influenced by key environmental factors such as temperature, food, salinity and light [18,19,21–24]. Temperature cues control differential reproductive energy allocation, favouring polyp budding at warm temperature regimes or triggering strobilation when cold temperature thresholds are reached [18,19,22,24]. Food supply has a positive effect on both polyp and ephyra production [18,19] while podocysts seem to be produced only when food supply is low and temperatures are typically high [18,25].

Due to the inherent taxonomical uncertainty, the interpretation and cross-comparison of ecological data referring to different *Aurelia* species is problematic. Investigations on *A*. *aurita* (*sensu lato*) showed different eco-physiological responses to environmental factors among distinct geographical *Aurelia* populations (e.g. [18,22,23]). Available evidence on the phylogenetic high diversity of the *Aurelia* group suggests these differences may be related to inter-specific genetic differences rather than intra-specific adaptive plasticity [16,26]. Therefore, to produce species-specific results on *Aurelia* jellyfish populations new experimental data should be associated with suitable taxonomic identification at species level.

Recently, an integrative morphometric and molecular approach helped in resolving taxonomic uncertainty around the moon jellyfish populations in the Mediterranean Sea, identifying three different species, namely the non-indigenous *A*. *coerulea* and *A*. *solida*, and the native *A*. *relicta* [27].

In this study, we analysed the combined effects of three key environmental factors—temperature, salinity and food supply—on polyp reproduction of two species, *A*. *coerulea* and *A*. *relicta*, from two different coastal habitats located at two opposite sides of the Adriatic basin: the Varano lake (Italy) and the Mljet lake (Croatia). The aim of this work was to obtain a better understanding of the biological mechanisms supporting the spatial separation and local population success of two different *Aurelia* species (one native and one non-native of the Mediterranean sea) in two spatially and ecologically distinct habitats, and to analyse their inter-specific eco-physiological differences.

## Materials and methods

### Ethics statement

The moon jellyfish *Aurelia coerulea* and *A*. *relicta* are not endangered or protected species and they are renowned as outbreak-forming invertebrate species with a high regeneration potential. Permit of sampling A. relicta in the protected area of the Big Lake of Mljet was kindly provided by the Natural Park of Mljet (Croatia). No permits were needed for sampling in the lake of Varano (Italy) adult *A*. *coerulea* medusae brooding planula larvae, which gave origin to the corresponding experimental polyp group.

### Locations

The polyps originated from two semi-enclosed coastal sounds with limited contact with the open sea in the Adriatic: the Varano coastal lake (Italy: 41°52' N, 15°44' E), inhabited by a dense population of *A*. *coerulea* [27], an invasive alien species, and the marine lakes of Mjlet (Croatia: 42°46' N, 17°21' E) inhabited by the endemic *A*. *relicta* [14,27]. These two marine lakes are characterized by contrasting hydrological features determined by their geomorphological differences.

The Lake of Varano, about 65 km^2^ in surface area, is the largest coastal lake in Italy. It is classified as a lagoon, and is located on the Italian east coast, separated from the Adriatic Sea by a 10 km long land stripe with two small artificial canals at the extremities. The mean depth is 3.5 m with a maximum at 6 m. The water temperature ranges between 6.7 and 30.2°C and the salinity varies between 21.6 and 35.0 ppt [28]. The bottom waters are generally saltier and warmer. This is due to the strong evaporation and the input of fresh water that flows over the salty-warm water. The water exchange between the lake and the sea, which is primarily driven by the semi-diurnal tide, is weak and the water residence time is 1.5 year. The Lake of Varano is an oligo-mesotrophic ecosystem [28].

The Mljet lakes, Veliko Jezero (Big Lake) and Malo Jezero, are monohaline marine lakes located in the southern Adriatic island of Mljet, Croatia. These lakes are moderately eutrophic ecosystems (29). The Big Lake has an area of 1.45 km^2^ and a maximum depth of 46 m. During summer there is a strong thermocline at 15–20 m depth and the temperature varies between the constant minimum of 11°C in the bottom layers and 28°C in surface. The salinity ranges mainly between 37.5 and 38 ppt [29]. The surface exchange of water with the open Mediterranean Sea is weak, driven by a 1 km long, 10 m wide and 3.8 depth channel. These lakes are therefore virtually isolated from the Adriatic Sea [29–31].

The polyps of the two species live in contrasting environmental temperatures. In the Mljet lake, the polyps of *A*. *relicta* are found at depths (≤ 20 m) below the summer thermocline, at temperatures usually ranging between 11–20°C throughout the year [29, 30]. In contrast, polyps of *A*. *coerulea* in the Varano lake are exposed to a wider range of temperatures (6–30°C [28]). The experimental conditions were chosen to reflect as much as possible the natural conditions of each polyp group. Therefore, only *A*. *coerulea* polyps—living in the transitional habitat of the Varano coastal lagoon (under fluctuating salinity values) were studied under two different salinity values (24 and 37 ppt).

### Experimental design

All polyps were kept in separate microcosms in order to study their individual responses. Before the experiment, all polyps were kept at salinity 37. One month prior to the experiment, *Aurelia coerulea* and *A*. *relicta* polyps were individually transferred to a total of 270 microcosms and incubated under different salinity/temperature conditions and feeding treatments (Table 1), in order to acclimate to the experimental conditions. These conditions were chosen to reflect the variations experienced by the polyps in their natural environments. The microcosms consisted of small polystyrene flasks (50 ml) filled with 30 ml of 1.2-μm filtered seawater. A 12:12 light-dark cycle, typical of winter/spring light conditions [23] was set up. The polyps were individually fed with newly hatched *Artemia salina* nauplii (dry weight: 2.3 μg ind^−1^ or 0.93 μg C ind^−1^; based on [32]) placed one by one in contact with the tentacles using a Pasteur pipette under a stereo microscope. A preliminary assay estimated that 10 nauplii was the mean number of prey captured and swallowed by each single polyp at each feeding session, corresponding to 9.3 μg C. Availability of a higher number of nauplii resulted in a variable number of non-captured prey. Ten nauplii were therefore provided as a standard food portion to each polyp throughout all experimental feeding sessions. The polyps were fed at three food regimes: once a week (F1), twice a week (F2) and three times a week (F3) and the water was replaced 2–3 hours after feeding with seawater at the same temperature and salinity conditions (Table 1). The number of new buds, podocysts and ephyrae were counted after each feeding session and then removed from the microcosm. In order to evaluate the somatic growth, the calix diameter was measured on the first and the last day of the experiment, for 6 polyps in every condition. Measurements were performed using a stereo microscope with a graduated eyepiece. If the calix was not round, the average of the maximum and the minimum dimensions was used.

**Table 1:** Experimental conditions.

Species	Location	Experimental conditions		
		Salinity (ppt)	Temperature (°C)	Feeding treatment (μg C ind^−1^ week^−1^)
*Aurelia coerulea*	Varano (Italy)	24 / 37	14 / 21	F1: 9.3, F2: 18.6, F3: 27.9
*Aurelia relicta*	Mljet (Croatia)	37	14 / 21	F1: 9.3, F2: 18.6, F3: 27.9

Experimental conditions to examine the effects of temperature, salinity and food supply on the asexual reproduction of *Aurelia coerulea* and *A*. *relicta* polyps.

Three-way ANOVAs were performed on the observations. Data for the *A*. *coerulea* polyp group were first analysed on their own and then merged with the *A*. *relicta* polyp group in order to compare results between species. All counted values were square root transformed and tested for the normality of the data (Shapiro-Wilk test) and the homogeneity of variance (Bartlett test). When these assumptions were not satisfied, additional non-parametric tests were carried out (Kruskal-Wallis) in order to allow the use of the ANOVAs results. When significant interactions were found between factors, two-way and one-way ANOVAs were carried out and implemented with interaction plots to verify whether the effect produced by a factor on the quantitative variable was due to interaction with another factor. All these tests were carried out using R-statistics 2.14.1.

## Results

### Budding

**Fig 1:**
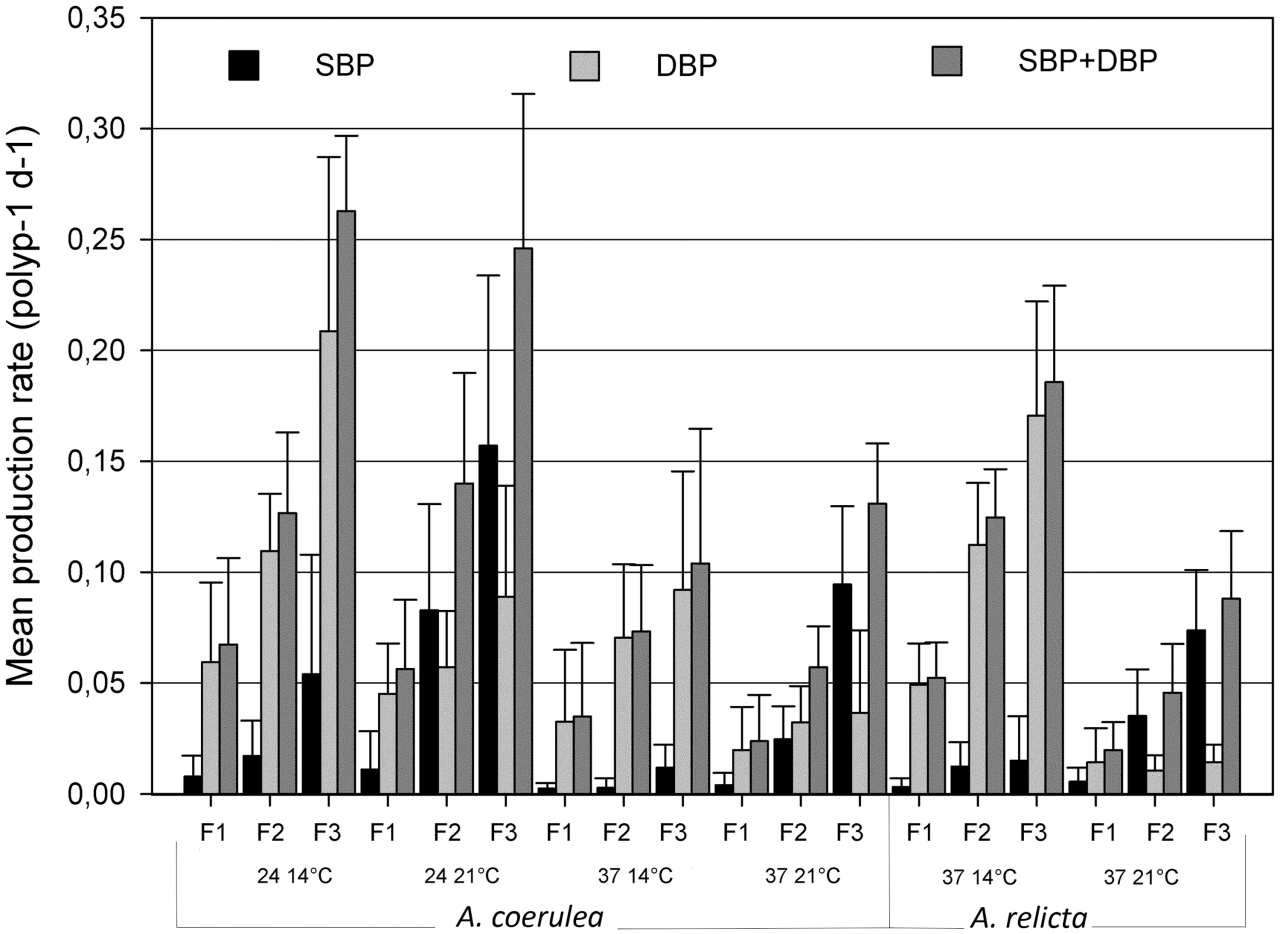
Budding rates. Mean budding rates and upper standard deviation from polyps of *Aurelia coerulea* and *A*. *relicta* under different combinations of experimental conditions over 85 days. F1, F2, F3 = Feeding regimes of 9.3, 18.6, 27.9 μg C ind^−1^ week^−1^, respectively; Salinity 24 or 37 ppt; Temperature 14°C or 21°C. DBP = Directly Budded Polyp, SBP = Stolonal Budded Polyp.

Polyps of both species produced buds under all experimental conditions at a nearly constant rate for each treatment (Fig 1). The total bud production rates increased with the food supply and were higher at 24 salinity for the *A*. *coerulea* polyp group. The effect on budding under temperature changes differed between the two species, with high significant effect only on *A*. *relicta* polyps. A highly significant stimulatory effect on the number of buds per polyp was observed under increasing feeding regimes in both species (*A*. *coerulea*: F (2, 168) = 223.21, P<0.0001; *A*. *relicta*: F (1, 158) = 130.39, P<0.0001; Table 2).

**Table 2:** Statistical results.

	Group	*A*. *coerulea*			Group	*A*. *coerulea vs*. *A*.*relicta*		
Dependent variable	Factor	*F*	df	p-value	Factor	*F*	df	p-value
**Total number of buds**	Food	223.21	2	0.000***	Food	130.39	2	0.000***
	Temperature	0.33	1	0.564	Temperature	26.42	1	0.000***
	Salinity	198.65	1	0.000***	Group	11.33	1	0.000***
	Salinity*Food	7.123	2	0.001**	Temperature*group	42.8	1	0.000***
	Group	***A*. *coerulea***			Group	***A*. *relicta***		
Dependent variable	Factor	*F*	df	p-value	Factor	*F*	df	p-value
**Number of DBP/SBP**	Budding mode	132.23	1	0.000***	Budding mode	80.45	1	0.000***
	Food	136.19	2	0.000***	Food	53.39	2	0.000***
	Temperature	4.22	1	0.041	Temperature	49.57	1	0.000***
	Budding mode*Temperature	180.43	1	0.000***	Budding mode*Temperature	245.17	1	0.000***
	Budding mode*Food	7.8	2	0.000***	Budding mode*Food	2.4	2	0.000
	Group	***A*. *coerulea***			Group	***A*.*coerulea vs*. *A*.*relicta***		
Dependent variable	Factor	*F*	df	p-value	Factor	*F*	df	p-value
**Number of podocysts**	Food	0.29	2	0.746	Food	0.95	2	0.3904
	Temperature	44.11	1	0.000***	Temperature	6.39	1	0.124*
	Salinity	12.22	1	0.000***	Group	2.4	1	0.000***
	Temperature*Salinity	15.93	1	0.000***				
	Group	***A*. *coerulea***			Group	***A*.*coerulea vs*. *A*.*relicta***		
Dependent variable	Factor	*F*	df	p-value	Factor	*F*	df	p-value
**Calyx Diameter growth**	Food	9.15	2	0.000***	Food	4.37	2	0.0169*
	Temperature	36.28	1	0.000***	Temperature	24.85	1	0.000***
	Salinity	0.64	1	0.426	Group	77.76	1	0.000***

Summary of three-way ANOVA run on experimental data collected from 270 polyps of *A*. *coerulea* and *A*. *relicta* to examine the effects of food, temperature, salinity on the total number of buds, the number of podocyst, the calyx diameter growth, or the effects of budding mode, food and temperature on the relative number of DBP/SBP. The use of *, **, and *** denotes levels .05, .01, and .001 of statistical significance, respectively.

**Fig 2:**
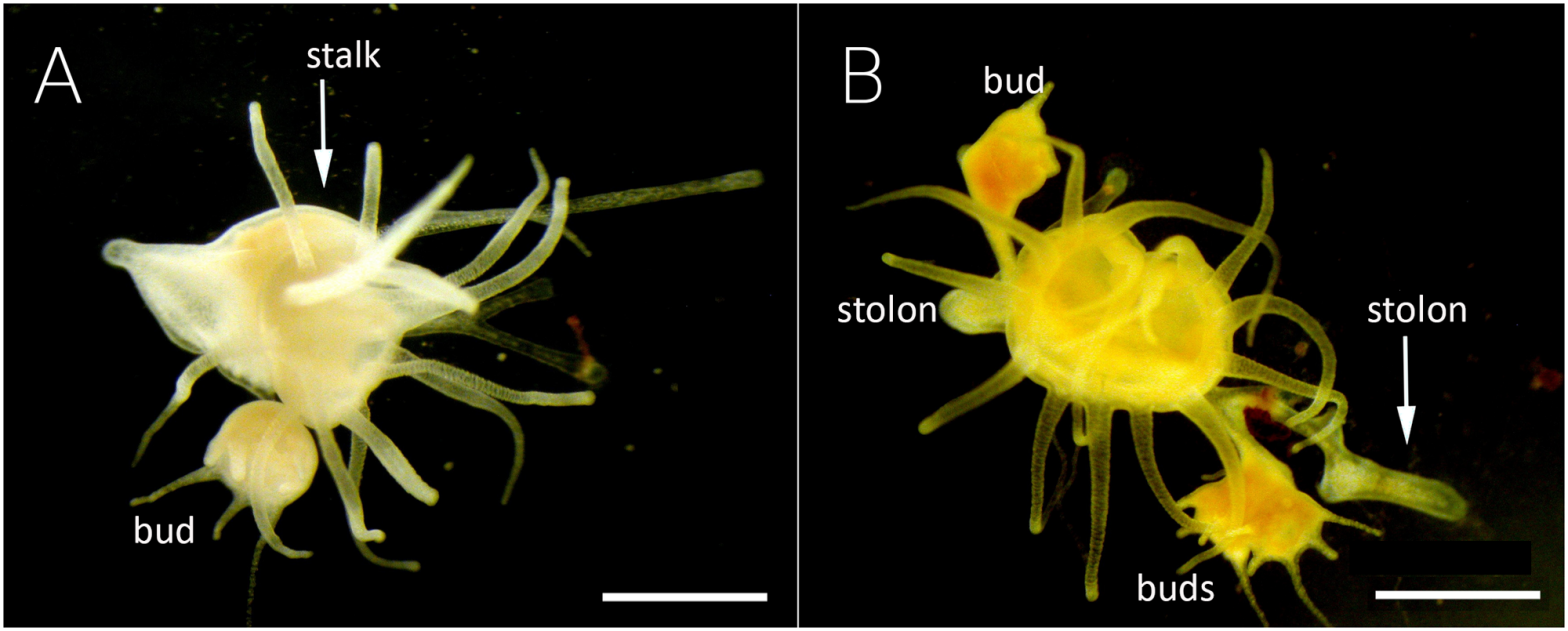
Budding modes. Budding modes of *Aurelia coerulea* and *A*. *relicta* polyps. (A) Polyp producing a Directly Budded Polyp (DBP) from the stalk; (B) polyp producing Stolonal Budded Polyps (SBP) from the pedal stolon. Scale bars: 1 mm.

The new budded polyps were counted in two categories: directly budded polyp (DBP) or stolonal budded polyp (SBP). The DBP are produced by the stalk of the parent and start to grow tentacles before detaching from the parent, using a pedal stolon (Fig 2A). By contrast, SBP start growing from the parent’s pedal stolon and the development of the new polyp does not start until the stolon has attached to the substrate (Fig 2B). For the 270 polyps incubated, the most common mode of budding was the direct budding. Indeed, 66% of the new polyps were directly budded (DBP).

As presented in Table 2, a highly significant difference was found between the 2 budding modes (*A*. *coerulea*: F (1, 348) = 300.59, P<0.0001; *A*.*relicta*: F (1, 168) = 80.45, P<0.0001) as well as a significant interaction between the budding mode and the temperature, for both populations (*A*. *coerulea*: F (1, 348) = 180.43, P<0.0001; *A*. *relicta*: F (1, 168) = 245.17, P<0.0001). Direct budding appears to be always favoured at 14°C (Fig 1) while at 21°C it is only favoured at the lowest feeding treatment (F1). Stolon budding was favoured at 21°C under high feeding treatment (F3). The interaction between budding mode and feeding treatment was highly significant [F (2, 348) = 7.80, P<0.0001] for *A*. *coerulea* polyps.

### Podocysts

**Fig 3:**
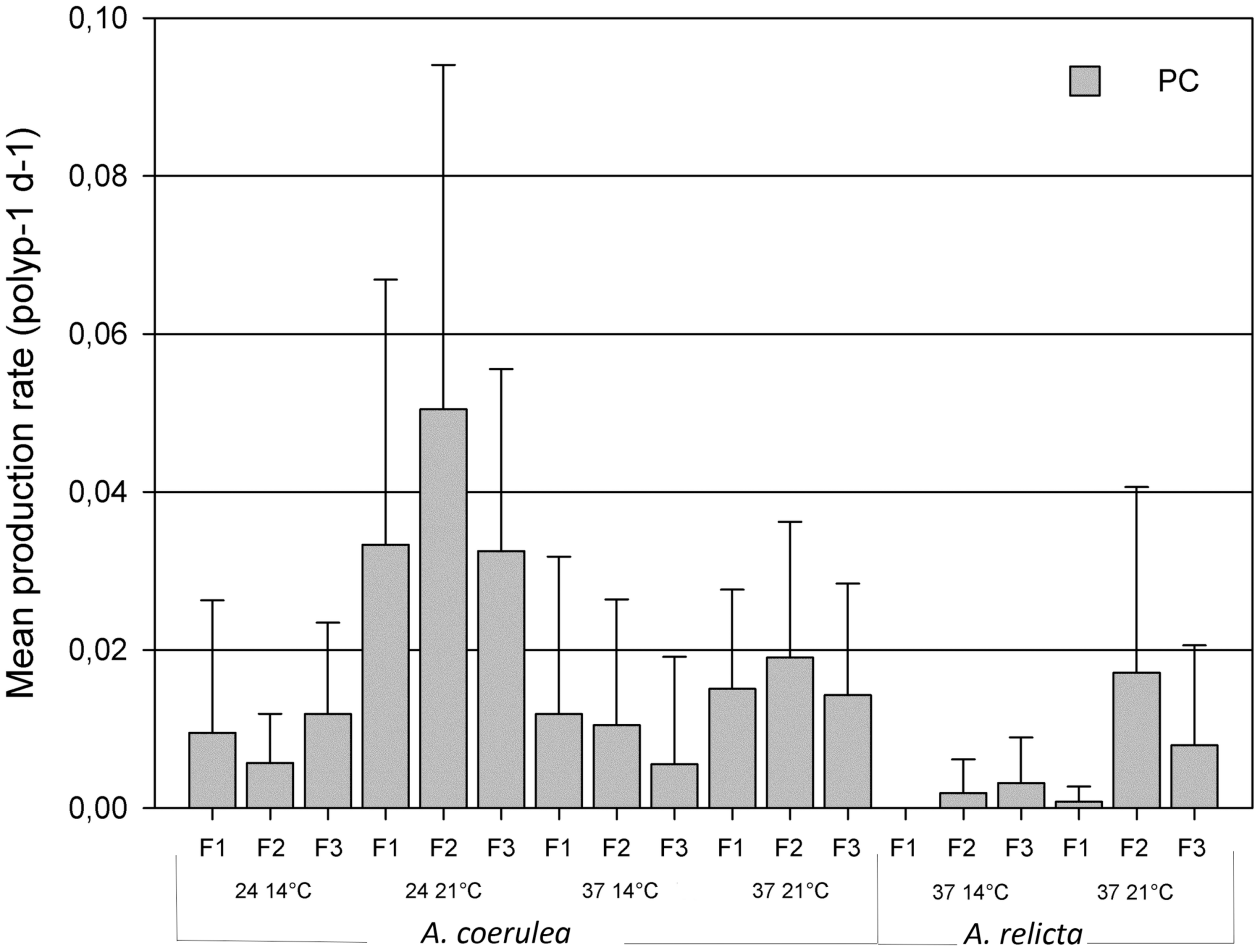
Podocyst production. Mean production rates of podocysts (PC) obtained from polyps of *Aurelia coerulea* and *A*. *relicta* under different combinations of experimental conditions over 85 days. F1, F2, F3 = Feeding regimes of 9.3, 18.6, 27.9 μg C ind^−1^ week^−1^, respectively; Salinity 24 or 37 ppt; Temperature 14°C or 21°C.

Podocysts were produced in most conditions, except for the *A*. *relicta* polyps maintained at 14°C under the F1 feeding regime (Fig 3). Nonetheless, not every single polyp produced podocysts. The production rates were higher at 21°C, with the *A*. *coerulea* polyps showing the highest podocyst production frequency at salinity 24, 93% of polyps (i.e., 14 out of 15) against 60% of polyps (i.e., 9 out of 15) at salinity 37. In the same conditions, the *A*. *relicta* polyps produced fewer podocysts than the *A*. *coerulea* polyps.

The number of podocysts produced per *A*. *coerulea* polyp was highly significantly influenced by temperature (F (1, 168) = 44.11, P<0.0001), salinity (F (1, 168) = 12.22, P<0.0001) and by the interaction between temperature and salinity (F (1, 168) = 15.93, P<0.0001) (Table 2). The number of podocysts produced per *A*. *relicta* polyp was significantly influenced by temperature (F (1, 168) = 180.43, P<0.01) (Table 2). Overall, a highly significant difference was found between the podocyst productions of the two *Aurelia* polyp groups (F (1, 168) = 20.80, P<0.0001).

### Somatic growth

**Fig 4:**
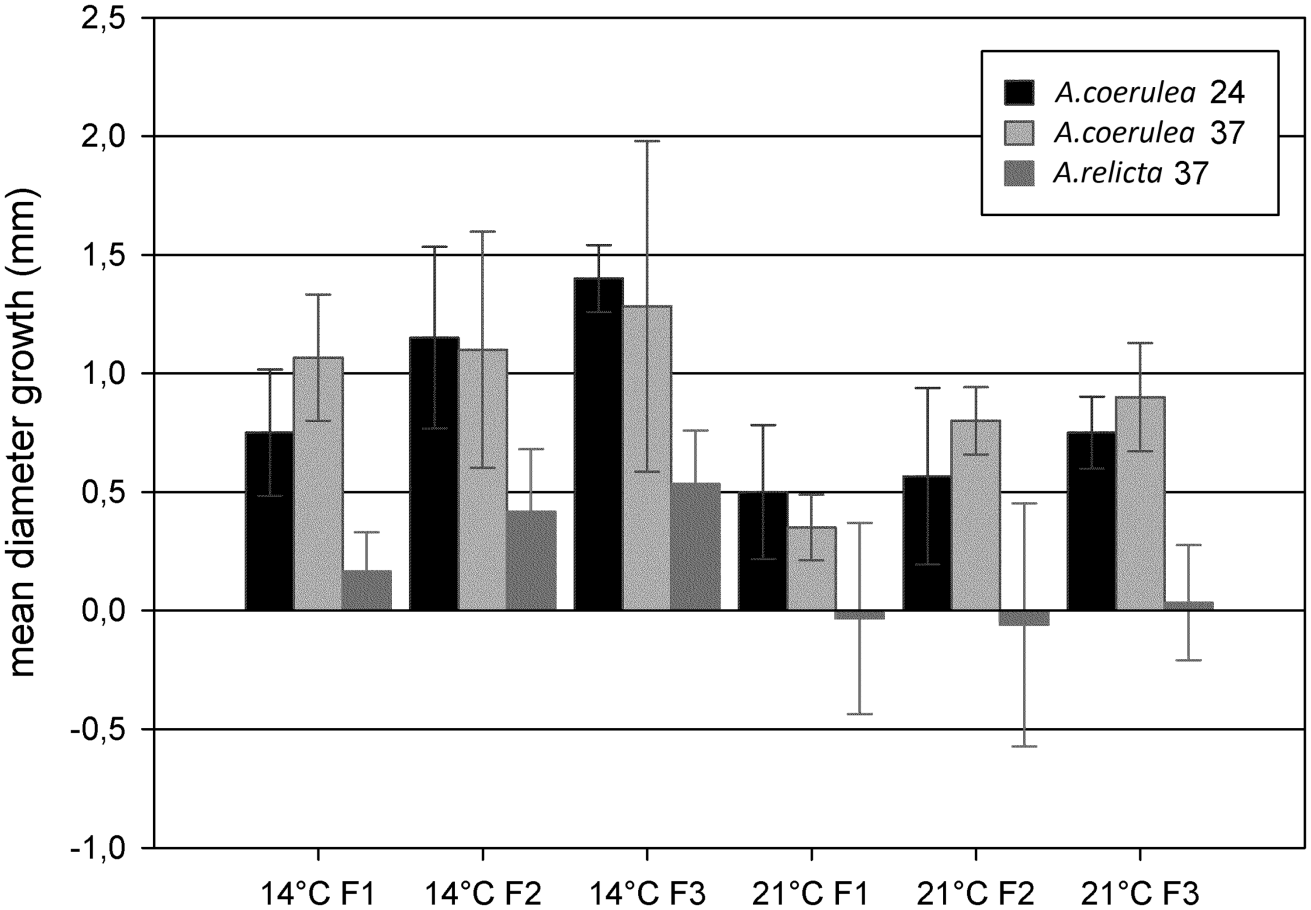
Calyx diameter increase. Comparison of means of calyx diameter increase of the *Aurelia coerulea* and *A*. *relicta* polyps incubated under different combinations of experimental conditions over 85 days. F1, F2, F3 = Feeding regimes of 9.3, 18.6, 27.9 μg C ind^−1^ week^−1^, respectively; Salinity 24 or 37 ppt; Temperature 14°C or 21°C. Vertical lines: SD.

Throughout the experiment, the calyx diameters of *A*. *coerulea* polyps were larger than those of *A*. *relicta* polyps (Fig 4). As expected, the highest feeding regime (F3) led to a proportionally higher increment of polyp size in both populations. Both polyp populations achieved larger size increases at 14°C than at 21°C. Highly significant differences in polyp size were detected in *A*. *coerulea* group according to feeding treatment (F (1, 60) = 9.15, P<0.0001) and temperature (F (1, 60) = 36.28, P<0.0001), but the effect of salinity was not significant (F (1, 60) = 0.64, P = 0.426). Results from *A*. *relicta* polyps showed a significant effect of feeding treatment on the somatic growth (F (1, 59) = 4.37, P<0.05) and a highly significant effect of temperature (F (1, 59) = 24.85, P<0.0001). A highly significant difference (F (1, 59) = 77.76, P<0.0001) between the *A*. *coerulea* and the *A*. *relicta* polyps was observed, with the latter growing less than the former ones (Fig 4). Further, the *A*. *relicta* polyps at 21°C exhibited degrowth, i.e. decrease in calyx size with respect to initial conditions.

### Strobilation and polyp regression

**Fig 5:**
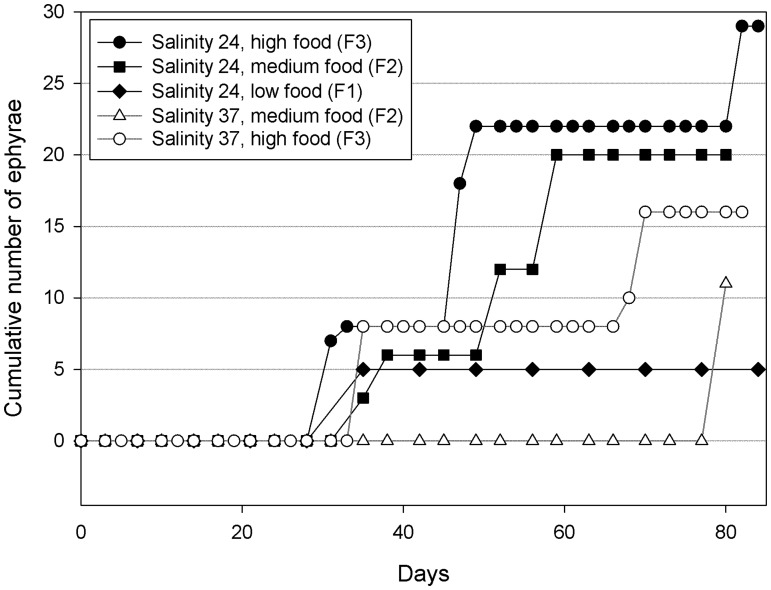
Ephyrae production. Cumulative number of ephyrae produced by *A*. *coerulea* polyps at 14°C under different combination of salinities and food regimes (F1, F2, F3 = food regimes of 9.3, 18.6, 27.9 μg C ind^−1^ week^−1^, respectively; Salinity 24 or 37 ppt). No ephyrae were produced by *A*. *relicta* polyps.

Strobilation leading to liberation of ephyrae was low and only observed at 14°C in the *A*. *coerulea* polyps, whereas no ephyrae were produced by the *A*. *relicta* polyps. Overall, the low number of produced ephyrae prevented the application of statistical tests. However, it was noticed that the number of strobilae and the number of ephyrae produced per polyp in the *A*. *coerulea* group increased with the feeding regime (Fig 5). Conversely, the low production of ephyrae at salinity 37 suggests a negative effect of high salinity on the *A*. *coerulea* group.

**Fig 6:**
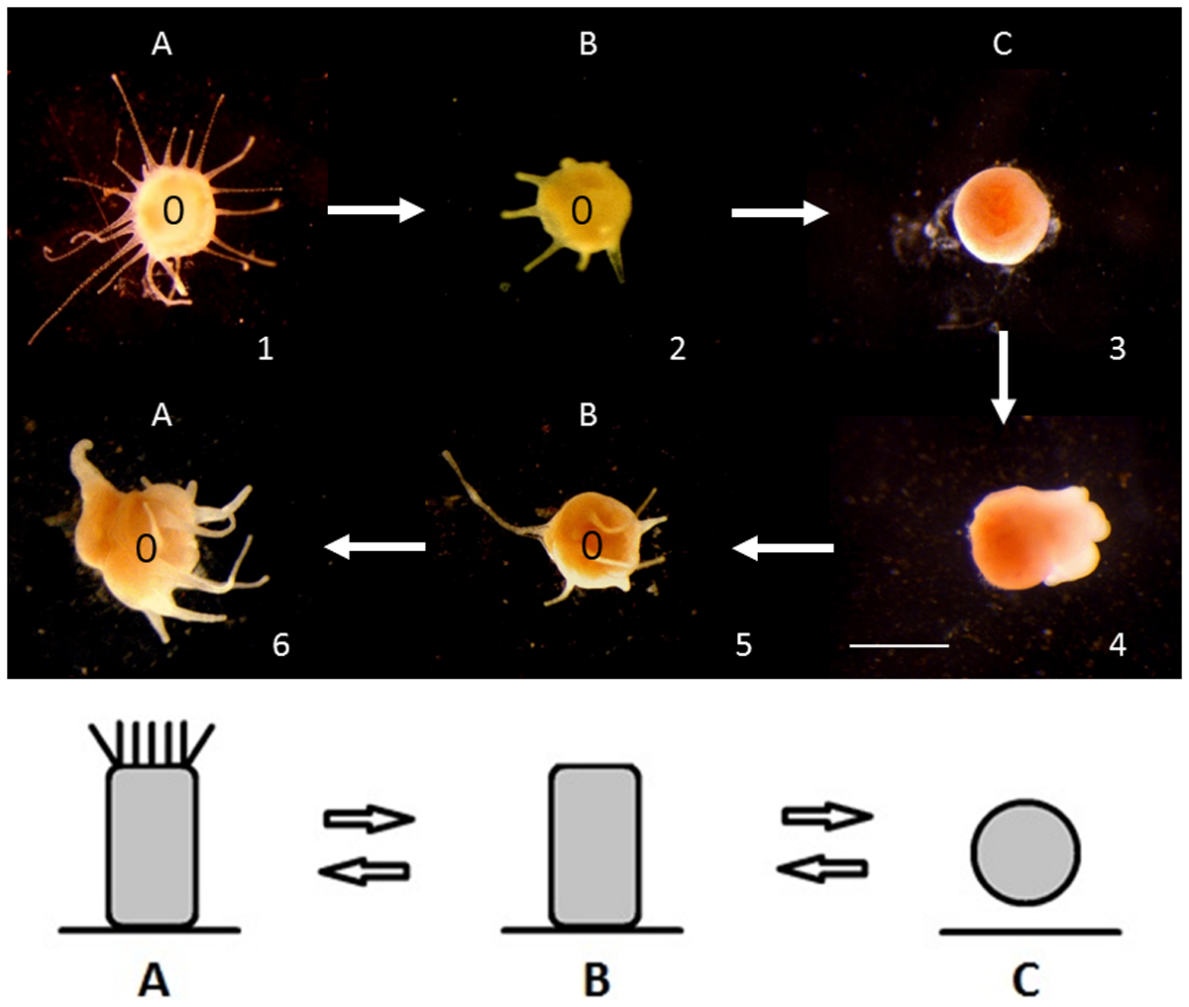
Regression-regeneration cycle. Regression-regeneration cycle of *Aurelia relicta* polyp. (A) fully active polyp, (B) polyp in transition, (C) unattached, drifting non-feeding stage. (o), oral side. Arabic numbers indicate the progression of the process. Scale bars: 1 mm.

The *A*. *relicta* polyps exhibited a previously unrecorded phenomenon. Overall, for the 3 feeding regimes at 21°C, 78% of polyps (i.e., 35 out of 45) degenerated—with complete resorption of tentacles, mouth, and pedal disk—each polyp transforming into a unattached, drifting non-feeding stage (RS) (Fig 6). After a dormant period, a fully differentiated, individual polyp with pedal disk, tentacles, mouth reformed from each non-feeding drifting stage while in the water column, then reattached to the bottom and became indistinguishable from a newly born polyp derived from planula metamorphosis. These polyps were able to repeatedly switch between the active and the resting phase. The increased feeding regime apparently increased the number of polyps entering a resting phase, i.e. from 60 (F1: 9 polyps out of 15) to 93% (F3: 14 polyps out of 15) of the experimental population. Conversely, no comparable regression stage was ever observed within the *A*. *coerulea* polyp group.

## Discussion

This study highlighted the differential influence of environmental variables (food supply, temperature, and salinity) on the efficiency and mode of asexual reproduction in the polyp stage of two *Aurelia* jellyfish species living at the opposite E-W sides of the Adriatic Sea, in two semi-enclosed coastal areas subject to remarkably different ecological conditions.

### Effect of food

Our results revealed a significant interaction between food supply and budding mode. For both species, the lowest food regime favoured direct budding (production of DBP) over stolonal budding (production of SBP). SBP usually remain closer to the parent polyp than the polyps originated by DBP. Such parental proximity may be disadvantageous at low food supply conditions. Indeed, the direct budding produce new polyps not immediately attached to the substrate, which are therefore susceptible to more movements or drifting by currents before final attachment. The spreading of DBP may eventually reduce competition for food among parental and newly budded polyps.

In contrast, for both species the differences of food supply regimes at the chosen experimental conditions did not determine significant variations on podocyst production. This finding corroborates previous observations suggesting that podocyst production occurs as a resistance strategy to poor food conditions [25]. In (undetermined) *Aurelia* sp. polyps from Japanese waters, podocyst production was induced at food regimes ≤4.8 μg C polyp^-1^ day^-1^, equivalent to 33.6 μg C ind^−1^ week^−1^ [25]. In our experiments, the selected food regimes were always below that threshold (F 1, 2, 3 = [9.3, 18.6, 27.9 respectively] μg C ind^−1^ week^−1^), which may explain the occurrence of podocysts in most feeding treatments. Within these conditions, the lack of differences among food supply regimes on podocysts suggests that food availability has a qualitative, yes-or-no effect on podocyst production rather than quantitative. However, this should be tested in extra feeding conditions.

The effect of food regime observed on ephyrae production from *A*. *coerulea* polyps showed that the number of ephyrae per polyp increases when food is more abundant, confirming previous results [19,33]. As a corollary, it is reasonable to postulate that an increase in zooplankton prey availability to polyp populations may induce larger blooms of medusae.

In both *A*. *coerulea and A*. *relicta* polyps, all ontogenetic alternatives such as somatic growth, budding, encystment, and strobilation are influenced by the available food supply. These mechanisms should be equally regarded as adaptive strategies common to Medusozoa to face with challenging environmental conditions, by alternatively entering a resting phase at low metabolic cost or by the liberation of ephyrae, free-living dispersal stage to escaping temporary disadvantageous habitat [34,35].

Food availability also controls the activation of a previously unknown resting mode, i.e. the morphological regression of the *A*. *relicta* polyps from the lake of Mljet. This process makes possible the alternation of feeding and non-feeding stages. The results imply that under warm temperature (21°C), a previously well-fed polyp has a better capacity to trigger the dormant cycle. Therefore, zooplankton prey may enhance the ability of polyps to enter a quiescent phase and thus increase their ability to face with subsequent prolonged stressful conditions.

Food supply is one of the key factors governing asexual reproduction and its effect on reproductive processes can be quantitative (e.g. budding rates, ephyrae production, somatic growth and quiescence capacity) and/or qualitative (e.g. onset or interruption of podocyst production, budding, strobilation, selection of budding mode, triggering dormant cycle). In this framework, we can hypothesize that increases in zooplankton abundance (due to e.g. climate-related changes of temperature or salinity, or global climatic oscillations; eutrophication, overfishing of planktivorous fishes) may ultimately lead to higher frequency and severity of jellyfish blooms.

### Effect of temperature

Polyps of *Aurelia relicta* can be found in the lake of Mljet at depths where temperatures are between 11–20°C throughout the year [29, 30], whereas polyps of *A*. *coerulea* in the Varano lake are exposed to a wider range of temperatures, from 6 to 30°C [28]. Accordingly, the polyp asexual growth and reproduction of the two species are differently influenced by temperature. The *A*. *coerulea* polyps were able to keep stable their total bud production at both experimental temperatures (14°C, 21°C), which is consistent with the optimal budding temperature for *Aurelia* spp. known to occur in the range 13–25°C [20–23]. Conversely, the budding of *A*. *relicta* polyps was negatively affected at 21°C. In addition, Han & Uye [18] observed in *Aurelia* sp. polyps from Japan an increase in budding production with temperature warmer than 25°C. Therefore, in spite of being often referred to a supposed large phenotypical plasticity of *A*. *aurita*, such wide range and differences in optimal temperatures reflect the existence of multiple cryptic *Aurelia* species and their peculiar adaptation to different conditions [26,27].

The identification of a significant interaction between the budding mode and temperature represents a key finding. Our results showed that polyps allocate more energy towards stolonal bud production (SBP) at warm temperature (21°C) and intermediate food availability (two feeding sessions per week, ≤18.6 μg C ind^−1^ week^−1^). This change in budding mode appears stronger for *A*. *relicta* polyp group.

This effect of temperature on budding mode does not agree with the results of Han & Uye [18] obtained with *Aurelia* sp. polyps from Japan reared at similar or higher food supply (11.9, 23.1, 46.2, 70 and 93.1 μg C ind^−1^ week^−1^) and temperature (18, 22, 26 and 28°C). Their experiments showed that the production of direct budded polyps (DBP) was the major mode in every treatment (94% of total buds). Altogether, these results corroborate the hypothesis that different *Aurelia* spp. may show wide intra- and inter-specific plasticity in terms of eco-physiological acclimation or evolutionary adaptive responses to environmental conditions. The genus *Aurelia* is known as one of the most specious taxa among Scyphozoa: for this reason, integrating morphological and molecular taxonomy will help to clarify the variability of ecophysiological responses within the *Aurelia* species complex [27].

In the field, *Aurelia* spp. strobilation occurs after polyps are exposed to a prolonged drop in temperature. A reduction of water temperature is known to trigger upregulation of one or more secreted proteins, which act as strobilation inducers in *A*. *aurita* polyps [36]. However, our results showed the production of ephyrae may occur without a drop in temperature. Indeed, limited strobilation was triggered in the *A*. *coerulea* polyps from the Varano lake maintained in the laboratory under constant low temperature (14°C). A similar observation was also reported by Holst [37] with North Sea *A*. *aurita* polyps maintained in the laboratory at 15°C for 22 months, but no explanatory hypothesis was provided. It might be worth noting that in both Holst's and our experiments, strobilation at constant temperature was observed only in winter, i.e. when polyp strobilation occurs in nature. This periodicity could be coincidental, but could also imply the presence of an internal biological rhythm.

Furthermore, *A*. *relicta* polyps from the lake of Mljet showed to be able to enter a temporary regression-regeneration process, morphological regression into an unattached and atentacled propagule, followed by the reformation of a tentacled feeding polyp. We suggest this process may be triggered by warm temperature: it was only observed at 21°C, unusual for *A*. *relicta* polyps, usually living in the Mljet lake under isotherms up to 19–20°C. In this free-drifting stage, the polyp may potentially survive unusual warmer temperatures, by escaping from unsuitable environmental conditions either sinking or drifting to more suitable (i.e. colder) depths or locations. Therefore, the drifting propagule can be seen as an additional strategy to the benthic podocyst as a stress-survival mechanism. These polyp-to-unattached propagule reversible transformation somehow recalls the asexual dispersive mechanism described by Vagelli as “gemmation” [38], who reported the internal and external production of free-swimming, ciliated planuloid propagules, either produced from the inner gastrovascular cavity or from the outer body surface of scyphistomae of *Aurelia aurita* (although taxonomic identification of the investigated species remained uncertain), following intense polyp feeding periods at optimal rearing conditions. In both cases these free-swimming propagules acted as dispersive stages, by means of a novel cloning mechanism adding to the well-known mechanisms of budding, podocyst formation, and strobilation. In all these processes, there is an increase of the final number of main forms of the life cycle (scyphistomae, ephyrae and medusae). In the case of the reversible transformation observed in *A*. *relicta*, the outcome is not linked to an increase in the total number of individuals, but merely to the survival of polyps occasionally exposed to unfavourable environmental conditions. Indeed, high temperature (21°C) has a significant negative effect on polyp somatic growth, particularly for the *A*. *relicta* polyps. In a previous study, Han & Uye [18] showed an inverse relationship between the somatic growth and the number of buds produced, explained by the body mass loss by the bud production. However, because *A*. *coerulea* polyps have similar total bud productivity at both experimental temperatures, the observed size decrease cannot only be explained by a higher bud production. We postulate that the podocyst production should also be considered as a body mass loss and therefore having a negative effect on somatic growth. In addition, subtle increases of temperatures are known to dramatically increase respiration rates [39]. This effect may cause important energy loss and thus should play an important role in the low somatic growth observed at the higher temperature. The stronger reduction in size observed within the Mljet polyps at 21°C indicates *A*. *relicta* is negatively affected by high temperatures, witnessing to be a stenothermal species with a more restricted acclimation ability than *A*. *coerulea*.

### Effect of salinity

Salinity is considered a key factor affecting physiological performances of estuarine organisms [40]. However, unlike temperature and food supply having direct effects on the reproductive processes of the *Aurelia* spp. polyps, salinity appears to have only an indirect and thus less conspicuous influence, enhancing the efficiency of different reproduction modes rather than acting as a trigger.

In *A*. *coerulea* polyps from the Varano lake, low salinity seems to be coupled to better physiological performances. The wide distribution of *A*. *coerulea* in marinas, estuaries, and coastal lagoons may be accordingly in agreement with reduced salinity preferences [20,27]. However, more experiments on the effect of salinity on polyp reproduction are needed to clarify the colonization and blooming potential of different cryptic species of *Aurelia* spp.

## Conclusions

The two habitats (the Varano lake and the Mljet lake) are characterized by contrasting hydrological features induced by their geomorphological differences. Indeed, the two sites show wide variations in salinity and temperature ranges. The *A*. *coerulea* polyps in the poly-haline lagoon of Varano face large seasonal variations, with salinity ranges between 22 and 32 ppt and temperatures oscillating between 7 and 30°C [28]. Conversely, the *A*. *relicta* polyps live in the mono-haline lagoon of Mljet at stable marine salinity (37) and under a narrower range of temperatures over the year (11–20°C) [29].

Assuming that a high budding rate is a response to favourable conditions, the best productivities have been observed under low salinity (24 ppt) for the *A*. *coerulea* polyps, and at cold temperature (14°C) for *A*. *relicta* polyps. Compared to latter group, the *A*. *coerulea* polyps are euryvalent, being adapted to a fluctuating transitional habitat, therefore more tolerant and less negatively affected by relatively warm temperature (21°C). Such adaptive plasticity allows them to maintain a stable budding production at the two temperatures. However, the increase of podocyst production suggests that warm temperatures may be somehow stressful to polyps of *A*. *coerulea*. As suggested by Scorrano et al. [27], the preferential lagoonal- or harbour-limited distribution of *A*. *coerulea* provides strong indication this species entered the Mediterranean Sea through aquaculture and/or boating vectors, and that its high eco-physiological acclimation potential represent the key for its invasion success worldwide. Further investigations on the eco-physiological tolerance and ontogenetic potentials of polyp stages may improve our knowledge on the mechanisms underlying the biogeographic distribution of extant representatives of the *Aurelia* species complex, facilitating design and implementation of management and mitigation measures against potential impacts of jellyfish blooms. Also, the concept of phylogenetic niche conservatism [41] assumes that closely related species, due to a longer evolutionary history in common, tend to be more similar in terms of ecological niche requirements than distantly related species [42]. This can be considered as a potential evolutionary driving force, with both morphology and ecological requirements as lineage-specific evolving traits, leading to divergence associated to reproductive isolation and, eventually, speciation. It may be difficult to establish whether niche differences represent mechanistic explanations or just consequences of lineage splitting and speciation. However, species-specific eco-physiological potentials (e.g. tolerance to low salinity or high temperature of transitional habitats) may be regarded as a source of additional evolutionary information, which, in combination with morphological and molecular data, may help to compare the degree of phylogenetic relatedness within closely related species, such as cryptic species complex.

## Supporting information

S1 Dataset Overall raw data used for statistical analysis. (XLSX)

S2 Dataset Data shown in Figs 1 and 3. (XLSX)

S3 Dataset Data shown in Fig 4. (XLSX)

S4 Dataset Data shown in Fig 5. (XLSX)
